# Impact of COVID-19 outbreak on healthcare workers in a Tertiary Healthcare Center in India: a cross sectional study

**DOI:** 10.1038/s41598-023-50317-8

**Published:** 2024-01-17

**Authors:** Shahzad Mirza, V. R. Arvinden, Mercy Rophina, Jitendra Bhawalkar, Uzair Khan, Bhavin Chothani, Shivankur Singh, Tanya Sharma, Aryan Dwivedi, Ellora Pandey, Shivam Garg, Sahjid Sadrudin Mukhida, Zeeshan Shabbir Ahmed Sange, Shalini Bhaumik, Jessin Varughese, Vishwamohini Yallappa Devkar, Jyoti Singh, AnjuMol V. K., Veena K., Husen Shabbir Husen Mandviwala, Vinod Scaria, Aayush Gupta

**Affiliations:** 1 Dr. D.Y. Patil Medical College, Hospital and Research Centre, Dr. D.Y. Patil Vidyapeeth, Pimpri, Pune, Maharashtra 411018 India; 2 CSIR Institute of Genomics and Integrative Biology (CSIR-IGIB), Mathura Road, Delhi, 110025 India; 3 Academy of Scientific and Innovative Research (AcSIR), CSIR-HRDC Campus, Sector 19, Kamla Nehru Nagar, Ghaziabad, Uttar Pradesh 201002 India

**Keywords:** Viral infection, Risk factors, Fatigue

## Abstract

Numerous speculations have continually emerged, trying to explore the association between COVID-19 infection and a varied range of demographic and clinical factors. Frontline healthcare workers have been the primary group exposed to this infection, and there have been limited global research that examine this cohort. However, while there are a few large studies conducted on Indian healthcare professionals to investigate their potential risk and predisposing factors to COVID-19 infection, to our knowledge there are no studies evaluating the development of long COVID in this population. This cross-sectional study systematically utilized the demographic and clinical data of 3329 healthcare workers (HCW) from a tertiary hospital in India to gain significant insights into the associations between disease prevalence, severity of SARS-Cov-2 infection and long COVID. Most of the study population was found to be vaccinated (2,615, 78.5%), while 654 (19.65%) HCWs were found to be SARS-CoV-2 positive at least once. Of the infected HCWs, 75.1% (491) did not require hospitalization, whereas the rest were hospitalized for an average duration of 9 days. A total of 206 (6.19%) individuals were found to be suffering from long COVID. Persistent weakness/tiredness was the most experienced long-COVID symptom, while females (1.79, 1.25–2.57), individuals who consumed alcohol (1.85, 1.3–2.64) or had blood group B (1.9, 1.33–2.7) were at a significantly higher risk for developing long COVID.

## Introduction

The index case of COVID-19 was reported on January 30, 2020, in India^1^. This was followed by a sharp rise in the number of cases, leading subsequently to the adaptation of various combat strategies based on the population as well as the healthcare infrastructure of the nation^2^. Although most COVID-19 patients had mild to moderate symptoms, some patients, particularly those with underlying comorbid conditions and/or elevated levels of inflammatory markers, went on to develop serious disease and/or died^3^. Gradually, a handful of studies started reporting the presence of prolonged COVID-19 symptoms like fatigue, cough, shortness of breath, insomnia, mood disturbances, anxiety, and myalgias in a variable proportion of patients^4,5^. The World Health Organization (WHO) now estimates about 10–20% of infected patients suffer from “long covid”^6^.

Since its onset, India has reported over 43.2 million cases of COVID-19, making it the second most affected country in the world (following the USA). As of June 2021, the cumulative COVID 19 deaths in India were estimated to be more than 4 million^7^.

Healthcare workers (HCW), due to their frontline nature, have been at the greatest risk of contracting or spreading COVID-19 infection. The prevalence of infection among HCWs has exceeded 10% in Italy^8^, and a total of 9282 HCWs were confirmed with COVID-19 as of April 9, 2020, in the United States^9^. In India, the ICMR portal instituted to capture information of individuals undergoing testing for SARS-CoV-2 infection found 5% of HCWs to be positive for COVID-19 after an analysis of 21,402 records^10^. This cohort has been the focus of numerous studies post the SARS-COV-2 pandemic, most of which have focused solely on COVID-19 infection, its immediate sequelae and breakthrough infections (infection after vaccination)^11–14^, with few studies having a particular focus on long COVID symptoms^15–20^.

In the Indian context, there have been few studies evaluating the association of various clinical characteristics with the outcomes of COVID-19^21^, as well as studies assessing COVID-19 vaccination, amongst HCWs^22,23^.

However, a study that specifically evaluates long COVID in Indian HCWs is substantially lacking. This study, to our knowledge, serves as the first of its kind to perform a large-scale statistical analysis on a range of clinical and demographic characteristics of over 3000 HCWs in a tertiary care Indian hospital and reports rates of vaccinations, breakthrough infections, and reinfections (repeat SARS-CoV-2 infection) along with statistically significant predictors of long Covid.

## Materials and methods

### Study population and design

This cross-sectional study was carried out over a period of 12 weeks (July–October, 2021) after obtaining institutional ethical clearance, in accordance with the guidelines and regulations of the ethics committee. Informed consent was taken from all consultants, postgraduate and undergraduate students, interns, nurses, nursing auxiliaries, clerical staff, security personnel, administrative staff, pharmacists, and cleaners working in a tertiary care hospital that served as a dedicated 850 bed covid care hospital in western India. A 43-item survey (Supplementary Material) was designed using Google Forms and piloted to assess the design, feasibility, and validity of the questions. Data from the pilot was reviewed, and using feedback from experts, a definitive questionnaire was then used for the study. A team of postgraduate, undergraduate students and nurses conducted face to face interviews and filled in the form. Vaccination certificates as well as SARS-CoV-2 RT-PCR reports were manually verified, therefore, while most data in the study is patient-reported, details of infection positivity and vaccination were verified by medical records. For the purposes of this study, post COVID-19 symptoms, also known as “long COVID,” were defined as persistent symptoms 3 months from the onset of COVID-19 with symptoms that lasted for at least 2 months and could not be explained by an alternative diagnosis^24^. Adverse effects following immunization (AEFI) were defined as any untoward medical occurrence following immunization.

### Dataset used in the study

A questionnaire was used to collect a wide variety of information, which led to the aggregation of the study dataset. The study dataset comprised of patient-reported demographic and clinical information of over 3000 healthcare workers, including age, gender, height, weight, blood group, vaccination status, adverse effects following immunization (any untoward medical occurrence following immunization), severity of infection (which was categorized based on symptoms and the history of hospitalization, severe—needing ICU admission or intubation, moderate—general hospitalization and mild—symptomatic but under home isolation), history of hospitalization, long covid symptoms and lifestyle habits like smoking, use of tobacco, alcohol, diet, and other comorbidities if any.

### Estimation of statistical association

Univariate Fisher exact tests were performed to ascertain the association of long covid with various parameters (potential predictors) like age, sex, comorbidities (precisely including asthma, diabetes, hypertension, hyperlipidemia and hypothyroidism, stroke and tuberculosis), comorbidities, vaccination, reinfection, BMI index, and blood group. Multiple test corrections of p values were performed using the Bonferroni method. A multinomial regression model was generated for analysis of the severity of COVID-19 and its association with all the above-mentioned parameters using the multinom function from nnet (version 7.13-16)^25^ R package. p values were calculated from t statistics and multiple test corrections were performed. All data analysis and visualization was performed in R (version 4.1.2)^26^ using the ggplot2 package (version 2.3.3.5)^27^.

### Ethics declarations

This study was approved by the Institutional Ethics Sub-Committee (IESC) of Dr. D.Y. Patil Medical College Hospital and Research Centre (I.E.S.C/39/2021, Research Protocol No. IESC/FP/2021/33). The participants were explained about the informed consent process and the study was carried out as per the approved IESC guidelines, and in accordance with the Declaration of Helsinki.

## Results

### Data formatting and analysis

#### General demographic data

The study enrolled a total of 3329 HCWs belonging to over 20 departments. The mean age of the study group was 28.11 (± 8.95) years, with 2110 (63.38%) females and 1219 (36.62%) males. Undergraduate students (n = 849, 25.50%) and nurses (n = 822, 24.69%) comprised the major proportion of the dataset. Chefs and clerks were included under the administrative category and infection control staff in the nurse’s category. The majority of the participants had no history of smoking, tobacco chewing or alcohol drinking. Blood groups B (1043, 31.33%) and Rh positive (3003, 90.21%) were found to be the most common. 75.3% of the total enrolled were found to possess no clinical comorbidities, whereas asthma (57, 1.71%), diabetes (54, 1.62%), hypertension (64, 1.92%), stroke (1, 0.03%), hypo- and hyperthyroidism (68, 2.04%) were reported by the rest.

#### Vaccination status

**Figure 1:**
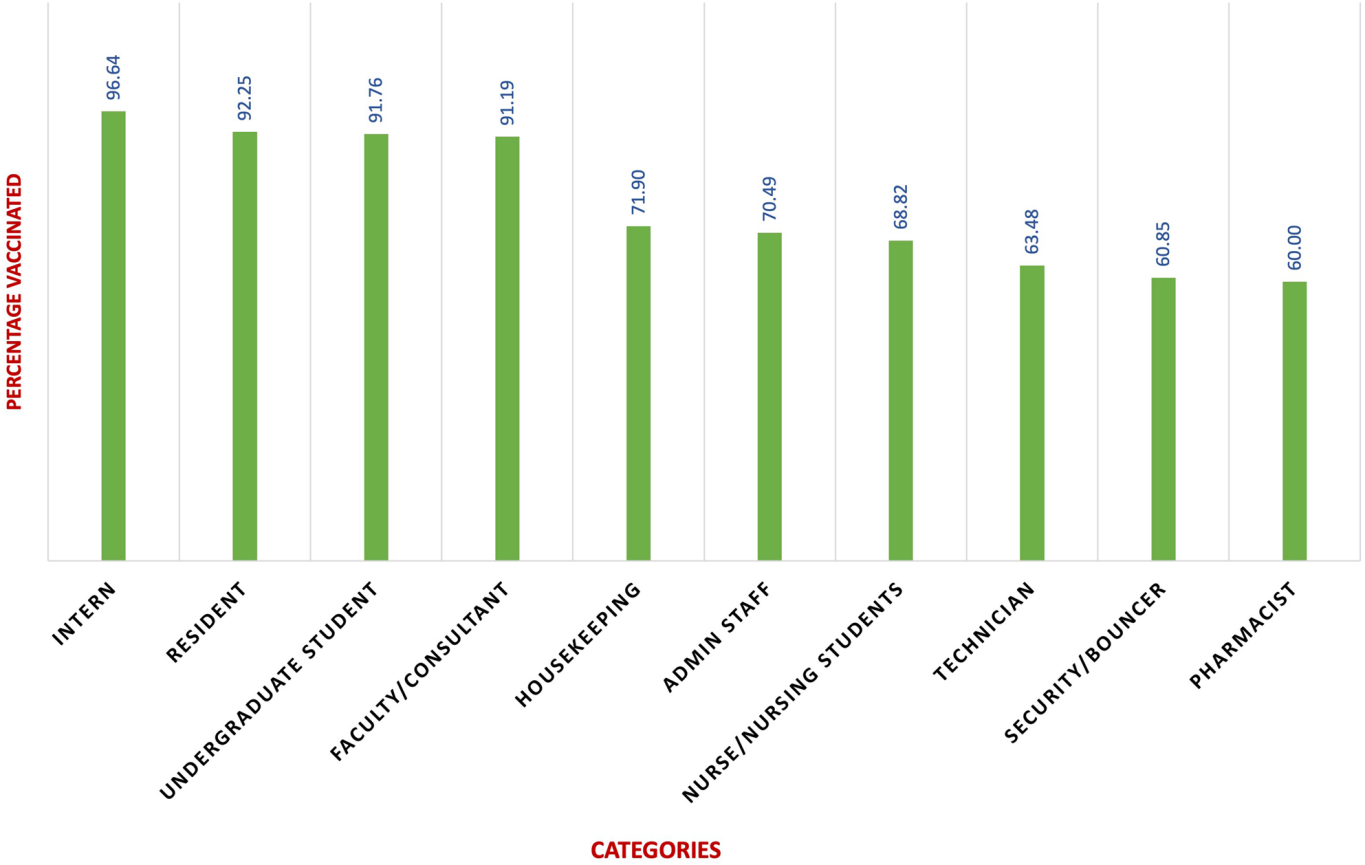
Schematic representation of the statistical association of vaccination status and occupational categories of healthcare workers enrolled in the study.

The study population found to be vaccinated at least once was 78.5% (2615), with the vaccines being Covishield (2293), Covaxin (303), Pfizer (7), Covovax (6), Sinopharm (5) or Sputnik (1), while the rest (714, 21.4%) were unvaccinated. There were multiple reasons reported for not taking a vaccine, including pregnancy, allergies, unavailability of slots and personal disinterest. Undergraduate students (91.76%), residents (92.61%), interns (96.64%) and faculty (91.19%) were found to be vaccinated while nurses (68.90%), technicians (63.48%) and other admin staff (69.13%) were vaccinated in lower percentages as compared with the prior groups as depicted in Fig. 1. No serious adverse effects following immunization (AEFIs) were observed in the current study.

#### Development of infection and severity of outcomes

**Figure 2:**
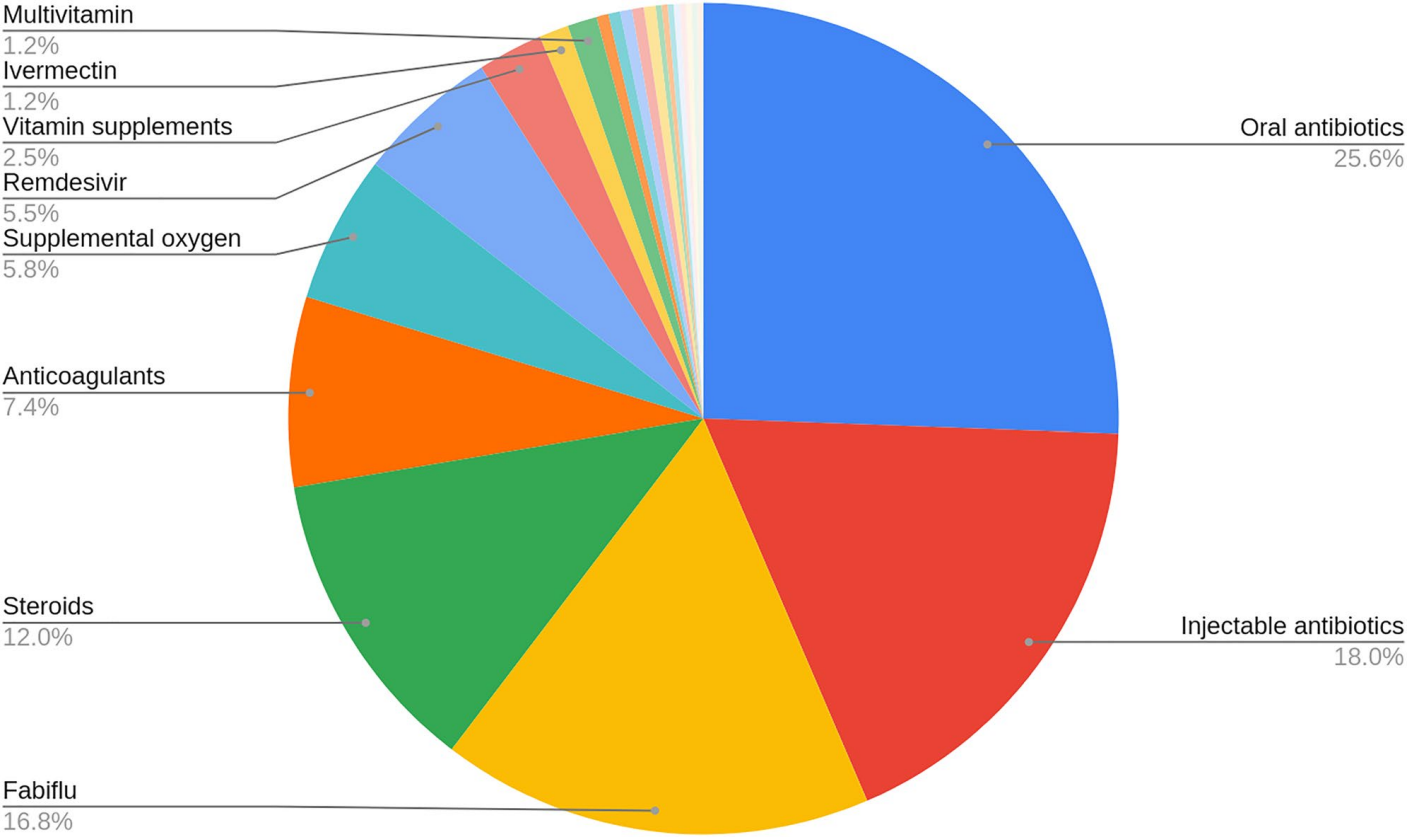
Schematic representation of the proportion of administration of various COVID-19 treatments.

This study found 654 (19.65%) HCWs to be SARS-CoV-2 positive at least once, of which a small proportion (98, 15%) did not get their infection confirmed by RT-PCR or antigen tests despite being symptomatic. Supplementary Table 1(A) provides a schematic representation of the odds of developing infection among various occupational categories of health care workers enrolled in the study. Fever, cough, body ache, breathing difficulties, and loss of smell and taste (symptoms typically observed during the delta wave) were the most reported symptoms. Of the infected HCWs, 75.1% (n = 491) did not require hospitalization, whereas the rest (24.6%, n = 161) were hospitalized for an average duration of 9 days. Oral and injectable antibiotics were the most administered treatments, followed by favipiravir, steroids, anticoagulants, supplemental oxygen, and remdesivir, as shown in Fig. 2. Based on symptoms and the history of hospitalization, infected patients were categorized as severe (needing ICU admission or intubation), moderate (general hospitalization) and mild (symptomatic but under home isolation). Of the total participants enrolled in the study, 55 (8.41%) were found to be asymptomatic, while 571 (87.31%), 23 (3.52%), and 5 (0.76%) experienced mild, moderate, and severe COVID-19 respectively. Although hypothyroidism and reinfection were found to be significantly associated with moderate and severe infections, owing to their minimal counts, the exact reliability of these results could not be ascertained.

A total of 165 (25.23%) cases had breakthrough infections of which 147 (single dose: 38, double dose: 109) took Covishield and 18 (single dose: 8, double dose: 10) took Covaxin. Symptoms due to breakthrough infections were largely mild with 16 and 145 individuals demonstrating asymptomatic or mild infections, while two each had moderate and severe disease. The mean time for breakthrough infection from the time of the first vaccine dose was found to be 61.89 days (range: 2 to 281 days). No significant difference was found between partially or fully vaccinated when breakthrough infection time was compared from the day of initial vaccination.

In addition, 25 (3.82%) cases of reinfection were also observed in our study population. Surprisingly, all reinfections were found in vaccinated individuals—6 (24%) partially vaccinated and 19 (76%) fully vaccinated. Further, 14 individuals with reinfection (56%) also reported the presence of long COVID symptoms.

### Overview of long covid analysis

The survey listed a range of long covid symptoms along with an open-ended option to allow us to capture unlisted symptoms, in keeping with the wide variability of symptoms. Two hundred and sixteen (216, 6.19%) individuals were found to be suffering from long COVID. Persistent weakness/tiredness, lasting from 12 weeks to 6 months, was the most experienced post-COVID symptom. Asthenia, loss of smell, myalgia, headache, neurotic symptoms, shortness of breath, loss of appetite, menstrual abnormalities, cough, joint pain, sore throat, frequent sleeping troubles, difficulties in concentration/confusion and leg pain were found to be the other common long covid symptoms. A handful of cases with rectal bleeding, weakness in eyesight, and panic attacks were also seen. The distribution of these symptoms is shown in Supplementary Fig. 1.

Surprisingly, undergraduate students (50%) who were attending most classes remotely for the study period (except for the in-person clinics/wards) were found to experience long covid symptoms more commonly, followed by nurses (14.08%) and residents (11.65%) as compared to the rest of the study group. The precise summary of the prevalence of long covid among various age groups and occupations is schematically shown in Supplementary Fig. 2.

**Figure 3:**
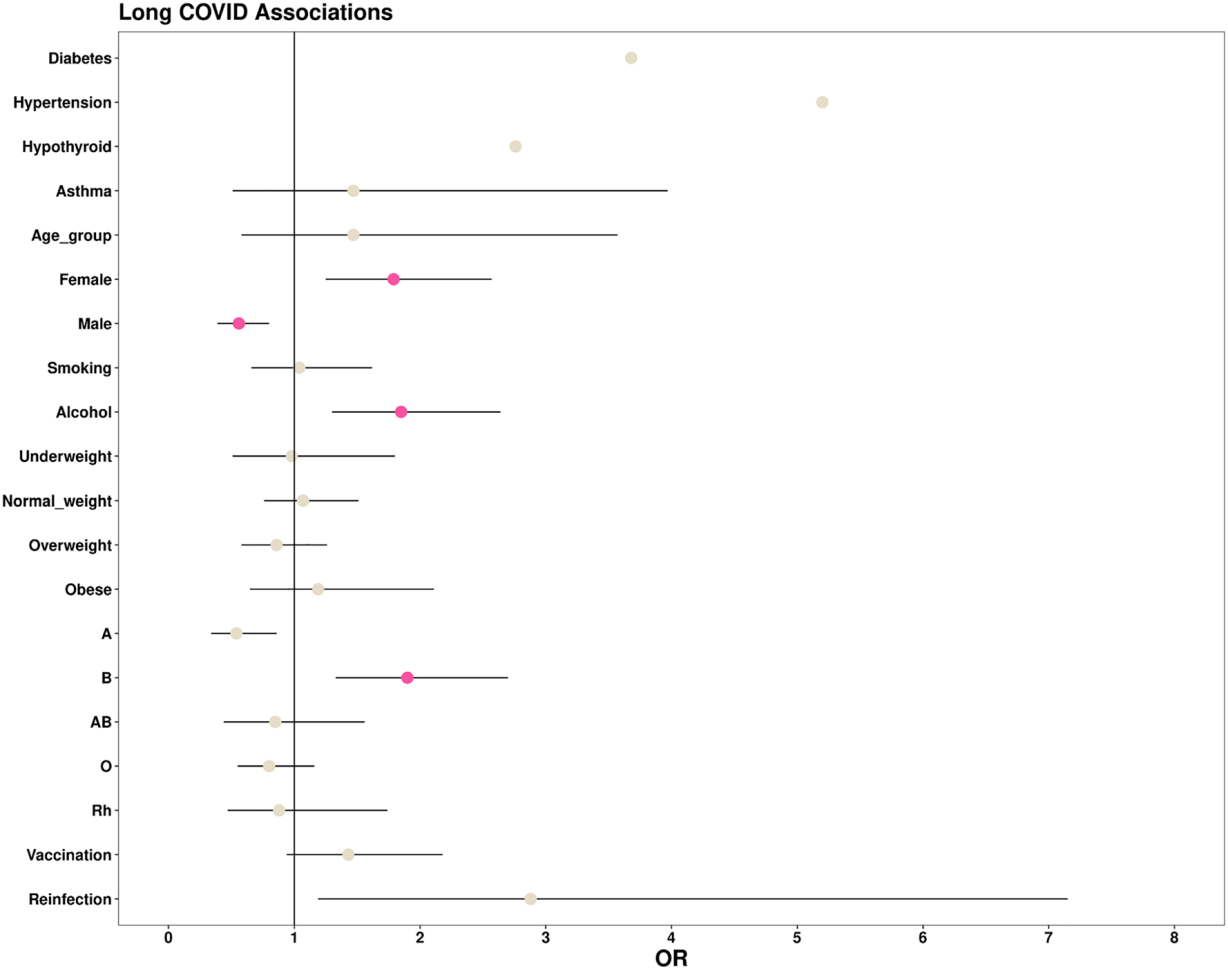
Schematic representation of statistically significant potential predictors and risk factors of long COVID.

We then tried to identify the presence of significant associations between long covid and various comorbid conditions, lifestyle factors, demographic factors, blood group, vaccination status, and history of infections. On univariate analysis, it was found that females possessed a significantly higher risk of long covid in comparison to males. In addition, drinking alcohol (22, 10.7%) and blood group B (65, 31.1%) were identified as significant risk factors for long covid in healthcare workers., as shown in Supplementary Table 2(B). A summarized tabulation of the demographic and clinical dataset is provided in Table 1, while Fig. 3 summarizes the statistically significant potential predictors and risk factors of long covid.

**Table 1:** Baseline clinical and demographic details of the study.

	Administrative	Faculty/consultant	Helper/attendant	Housekeeping staff	Intern	Nurses	Residents	Security	Technician	Undergraduate students	Total
Total enrolled count	142 (4.27%)	159 (4.78%)	357 (10.72%)	247 (7.42%)	119 (3.57%)	822 (24.69%)	284 (8.53%)	235 (7.06%)	115 (3.45%)	849 (25.50%)	3329
Number of males	69 (48.59%)	85 (53.46%)	139 (38.94%)	66 (26.72%)	40 (33.61%)	147(17.88%)	146 (51.41%)	147 (62.55%)	47 (40.87%)	333 (39.22%)	1219
Number of females	73 (51.41%)	74 (46.54%)	218 (61.06%)	181 (73.28%)	79 (66.39%)	675 (82.12%)	138 (48.59%)	88 (37.45%)	68 (59.13%)	516 (60.78%)	2110
Mean age	31.3	43.5	34.2	34.7	23.3	26.5	27.1	35.3	29.4	20.8	
Uninfected	112 (78.87)	121 (76.10%)	335 (93.84%)	233 (94.33%)	72 (60.50%)	712 (86.62%)	149 (52.46%)	218 (92.77%)	102 (88.70%)	621 (73.14%)	2675
Infected at least once	30 (21.31)	38 (23.90%)	22 (6.16%)	14 (5.67%)	47 (39.50%)	110 (13.38%)	135 (47.54%)	17 (7.23%)	13 (11.30%)	228 (26.86%)	654
Vaccinated	97 (68.31)	145 (91.19%)	249 (69.75%)	186 (75.30%)	115 (96.64%)	566 (68.86%)	262 (92.25%)	143 (60.85%)	73 (63.48%)	779 (91.76%)	2615
Unvaccinated	45 (31.69)	14 (8.81%)	108 (30.25%)	61 (24.70%)	4 (3.36%)	256 (31.14%)	22 (7.75%)	92 (39.15%)	42 (36.52%)	70 (8.24%)	714
Long haulers	4 (2.82)	19 (11.95%)	3 (0.84%)	4 (1.62%)	14 (11.76%)	30 (3.65%)	24 (8.45%)	3 (1.28%)	2 (1.74%)	103 (12.13%)	206
History of smoking	5 (3.52)	14 (8.81%)	18 (5.04%)	9 (3.64%)	32 (26.89%)	11 (1.34%)	73 (25.70%)	12 (5.11%)	3 (2.61%)	173 (20.38%)	173
History of tobacco chewing	4 (2.82)	3 (1.89%)	15 (4.20%)	11 (4.45%)	0 (0%)	3 (0.36%)	1 (0.35%)	26 (11.06%)	0 (0%)	0 (0%)	63
History of alcohol drinking	12 (8.45)	43 (27.04%)	35 (9.80%)	20 (8.10%)	51 (42.86%)	21 (2.55%)	147 (51.76%)	21 (8.94%)	6 (5.22%)	360 (42.40%)	716
Blood group—A	32 (22.54)	48 (30.19%)	77 (21.57%0)	36 (14.57%)	24 (20.17%)	222 (27.01%)	57 (20.07%)	53 (22.55%)	33 (28.70%)	173 (20.38%)	755
Blood group—B	44 (30.99)	46 (28.93%)	104 (29.13%)	69 (27.94%)	0 (0%)	269 (32.73%)	91 (32.04%)	68 (28.94%)	38 (33.04%)	314 (36.98%)	1043
Blood group—O	47 (33.10)	56 (35.22%)	93 (26.05%)	63 (25.51%)	30 (25.21%)	238 (28.95%)	109 (38.38%)	78 (33.19%)	30 (26.09%)	272 (32.04%)	1016
Blood group—AB	19 (13.38)	9 (5.66%)	83 (23.25%)	79 (31.98%)	65 (54.62%)	93 (11.31%)	27 (9.51%)	36 (15.32%)	14 (12.17%)	90 (10.60%)	515
Blood group—RHD+	110 (3.30%)	143 (4.30%)	292 (8.77%)	178 (5.35%)	109 (3.27%)	790 (23.73%)	268 (8.05%)	219 (6.58%)	107 (3.21%)	787 (23.64%)	3003
Blood group RHD−	8 (0.24%)	15 (0.45%)	8 (0.24%)	6 (0.18%)	10 (0.30%)	48 (1.44%)	16 (0.48%)	7 (0.21%)	6 (0.18%)	62 (1.86%)	
Comorbid conditions	12 (8.45)	49 (30.82%)	25 (7.00%)	26 (10.53%)	9 (7.56%)	33 (4.01%)	13 (4.58%)	11 (4.68%)	8 (6.96%)	49 (5.77%)	235

### Temporal trends of COVID-19 risk in healthcare workers

**Figure 4:**
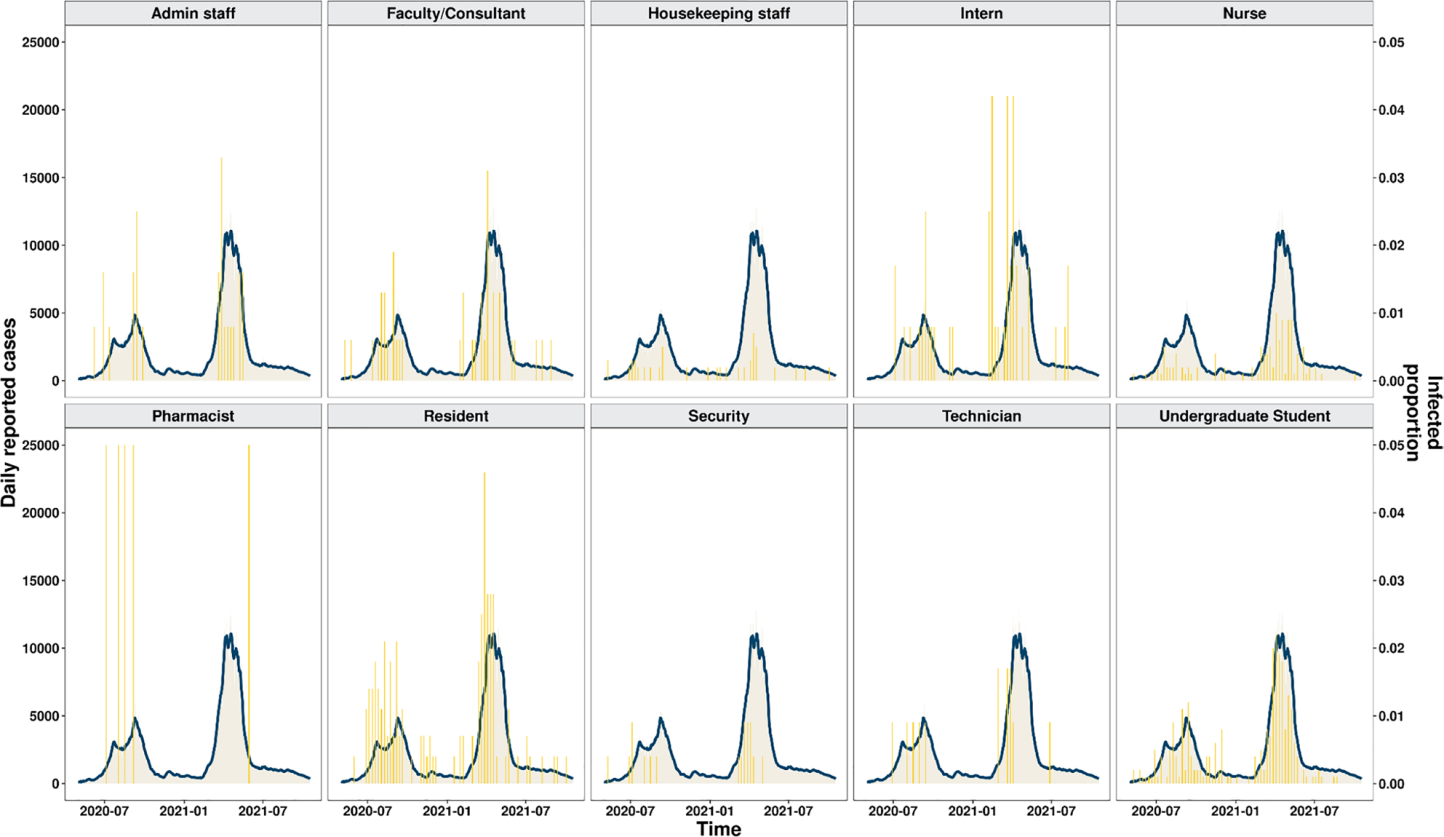
Graph representing the trend of cases observed in Pune, Maharashtra (the peaks in blue represent the overall cases reported in the city by occupation wise by https://www.covid19india.org/, while the bars in yellow represent the number of cases observed in each category in the study dataset).

To identify significant differences in the susceptibility to COVID-19 infections between the general population and healthcare professionals, a temporal trend analysis was performed to compare the infection rates reported in Pune from February 2020 to October 2021 with the infection rates estimated from the study dataset. Although our data was collected in 2021, the exact date of covid positivity and vaccination ranged from 2020, hence the overlap with the general population. Information on the daily reported COVID-19 cases from Pune was fetched from Covid19tracker.in. Our analysis revealed that there were no significant differences in the trend of COVID-19 onset between the general population and healthcare workers. An overview of the similarity observed in the infection rates is shown in Fig. 4.

## Discussion

Serious setbacks were faced by global healthcare systems due to the COVID-19 pandemic. Coronavirus cases in India increased dramatically in April 2021, reaching a record-breaking daily caseload of more than 40,000^28^. Indian healthcare workers experienced severe hardships due to the disparity between the number of physicians/healthcare facilities and the country's population^29^. Our study systematically utilized the demographic and clinical data of over 3000 HCW and found 19.65% of them to be SARS-CoV-2 positive at least once out of which 24.9% required hospitalization for an average duration of 9 days. Further, 6.19% of HCWs were found to be suffering from long COVID with persistent weakness/tiredness being the most experienced long-COVID symptom. According to the Indian Medical Association (IMA), about 87,000 HCWs contracted the infection, while 573 passed away due to it. Yet another study by the Indian Council of Medical Research (ICMR) suggested that due to staffing shortages in hospitals, 5% of the frontline HCWs, much lower than the present study (~ 20%), may have developed hospital-acquired COVID-19 infection (HAI)^30^.

This study explores the potential associations between various demographic and clinical factors with disease severity and post COVID syndromes among a large cohort of healthcare workers. Asthenia (abnormal physical weakness or lack of energy) emerged as the most common symptom among the long COVID symptoms observed. Per a few earlier reports, females were found to have a significantly higher COVID-19 risk than males^31,32^. A few studies have also found a strong correlation between human blood groups and COVID-19 severity and post-COVID symptoms. Blood group B, like previous studies, was found to possess an increased risk of predisposition to long COVID^32–34^.

Smokers were discovered to be more vulnerable to COVID-19 infection because of their immunocompromised lung health^35,36^. The Global Adult Tobacco Survey 2016–2017 found that 29% of Indian adults (15 years and older) used tobacco, making the country the second-largest consumer of tobacco products, making it a major risk factor for severe COVID^36^.

In addition to the above, possible associations of these factors with the severity of the illness were also investigated but owing to the limited numbers of severe infections in the study cohort, significant insights were not found.

## Conclusion

Although there exists some primary research exploring potential risk factors associated with COVID-19 infection outcomes and post-COVID symptoms, this study is among the handful that performs a large-scale cohort analysis of healthcare workers in India. This study found a total of 206 (6.19%) individuals to be suffering from long COVID with persistent weakness/tiredness as the most common symptom. The findings of this study supplement the existing evidence that healthcare workers are at an increased risk of infections (19.5%), breakthrough infections (25.23%) and reinfections (3.82%) along with the association of increased risk of long COVID with the female sex, alcohol intake, and blood group B.

### Supplementary Information

Supplementary Information.

## Data Availability

All data that support the findings of this study are available from the corresponding author upon reasonable request.
