# Supplementary material for: Impact of COVID-19 outbreak on healthcare workers in a Tertiary Healthcare Center in India: a cross sectional study
Source: Sci Rep. 2024 Jan 17;14:1504. doi: 10.1038/s41598-023-50317-8 (PMC10794462; doi:10.1038/s41598-023-50317-8)
Supplement: Supplementary file 1 — Supplementary Information. [file 41598_2023_50317_MOESM1_ESM.docx]

**Supplementary Figures**


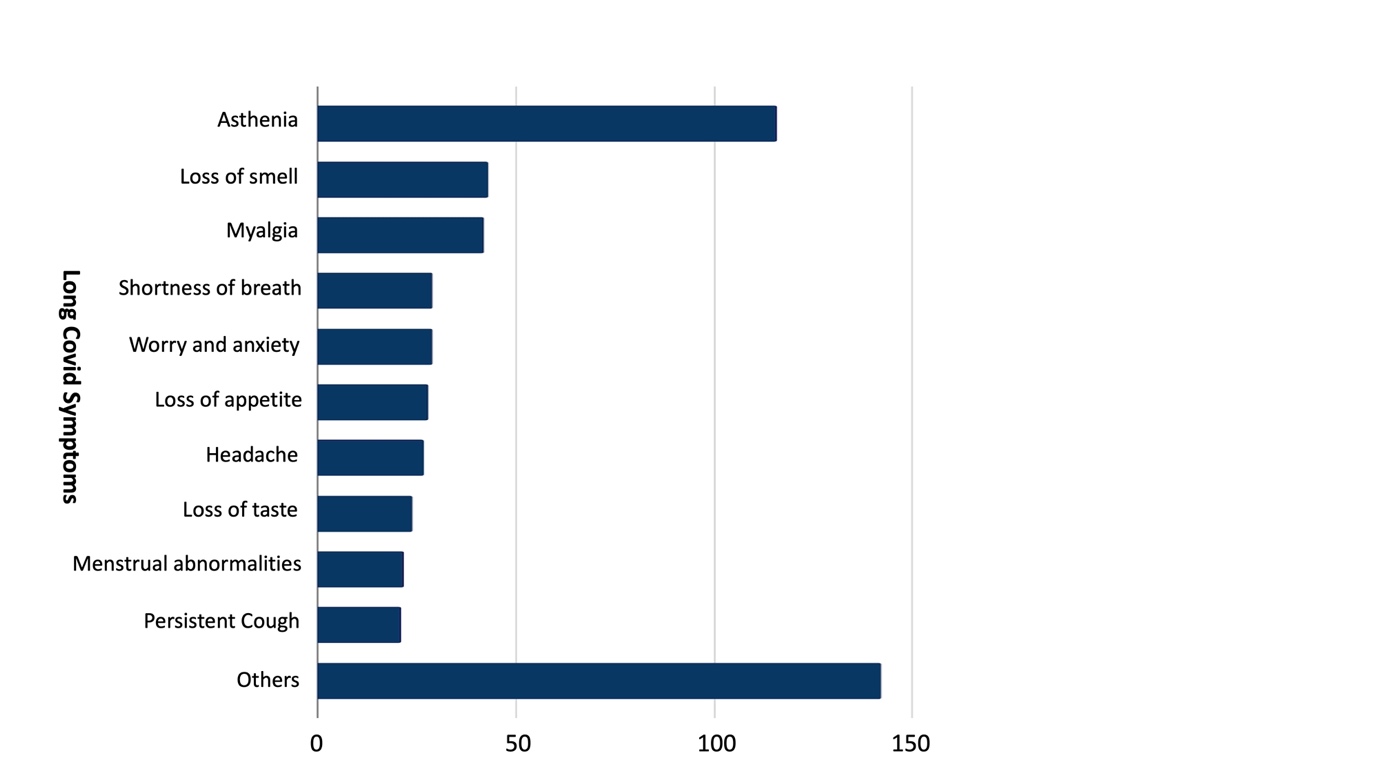


**Supplementary Figure 1.** Distribution of various long covid symptoms observed in the study.


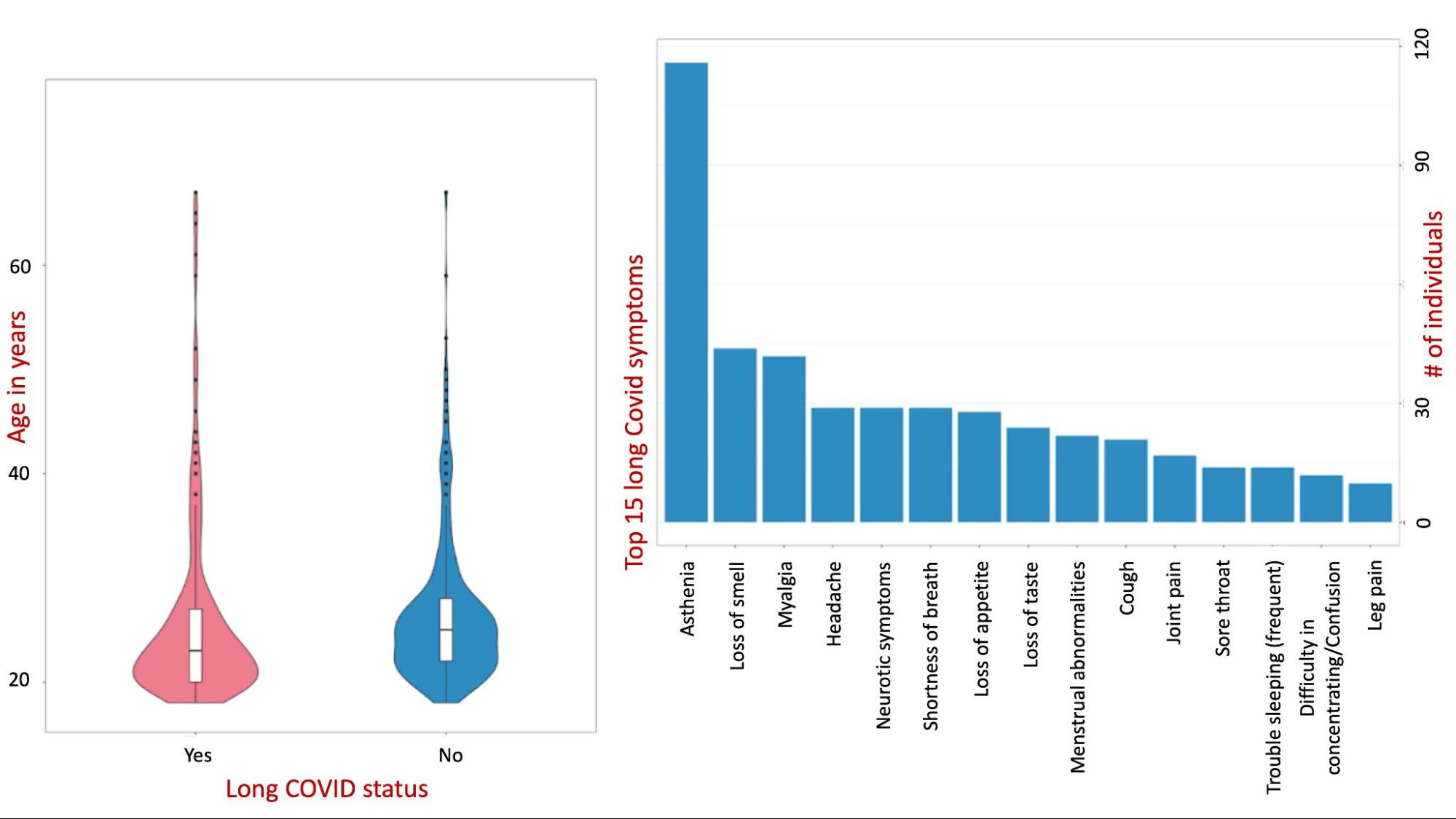


**Supplementary Figure 2.** Schematic representation of the status of long covid among various age groups and populations along with the highly prevalent long covid symptoms.

| **Category** | **p-value** | **OR** | **LOR** | **UOR** | **p-adj** | **Significant** |
| --- | --- | --- | --- | --- | --- | --- |
| Admin staff | 0.81 | 1.06 | 0.65 | 1.67 | 1 | Not-Significant |
| Housekeeping | 0 | 0.22 | 0.15 | 0.31 | 0 | Significant |
| Security | 0 | 0.3 | 0.17 | 0.5 | 0 | Significant |
| Technician | 0.02 | 0.51 | 0.26 | 0.92 | 0.22 | Not-Significant |
| Pharmacist | 0.57 | 1.37 | 0.39 | 3.97 | 1 | Not-Significant |
| Undergraduate student | 0 | 1.78 | 1.47 | 2.15 | 0 | **Significant** |
| Nurse | 0 | 0.55 | 0.44 | 0.69 | 0 | **Significant** |
| Intern | 0 | 2.8 | 1.87 | 4.14 | 0 | **Significant** |
| Resident | 0 | 4.41 | 3.4 | 5.71 | 0 | **Significant** |
| Faculty | 0.18 | 1.3 | 0.87 | 1.91 | 1 | Not-Significant |
|  |  |  |  |  |  |  |
|  |  |  |  |  |  |  |
| **Condition** | **p-value** | **OR** | **LOR** | **UOR** | **p-adj** | **Significant** |
| Diabetes | 0.11 | 3.68 | 0.71 | 23.94 | 1 | Not-Significant |
| Hypertension | 0.01 | 5.2 | 1.17 | 31.5 | 0.26 | Not-Significant |
| Hypothyroid | 0.14 | 2.76 | 0.59 | 14.04 | 1 | Not-Significant |
| Asthma | 0.46 | 1.47 | 0.51 | 3.97 | 1 | Not-Significant |
| Age group | 0.38 | 1.47 | 0.58 | 3.57 | 1 | Not-Significant |
| Female | 0.0009 | 1.79 | 1.25 | 2.57 | 0.0179 | **Significant** |
| Male | 0.0009 | 0.56 | 0.39 | 0.8 | 0.0179 | **Significant** |
| Smoking | 0.91 | 1.04 | 0.66 | 1.62 | 1 | Not-Significant |
| Alcohol | 0.0005 | 1.85 | 1.3 | 2.64 | 0.0106 | **Significant** |
| Underweight | 1 | 0.98 | 0.51 | 1.8 | 1 | Not-Significant |
| Normal weight | 0.73 | 1.07 | 0.76 | 1.51 | 1 | Not-Significant |
| Overweight | 0.45 | 0.86 | 0.58 | 1.26 | 1 | Not-Significant |
| Obese | 0.56 | 1.19 | 0.65 | 2.11 | 1 | Not-Significant |
| A | 0.00 | 0.54 | 0.34 | 0.86 | 0.11 | Not-Significant |
| B | 0.0002 | 1.9 | 1.33 | 2.7 | 0.0047 | **Significant** |
| AB | 0.66 | 0.85 | 0.44 | 1.56 | 1 | Not-Significant |
| O | 0.24 | 0.8 | 0.55 | 1.16 | 1 | Not-Significant |
| Rh | 0.75 | 0.88 | 0.47 | 1.74 | 1 | Not-Significant |
| Vaccination | 0.09 | 1.43 | 0.94 | 2.18 | 1 | Not-Significant |
| Reinfection | 0.01 | 2.88 | 1.19 | 7.15 | 0.27 | Not-Significant |

**Supplementary Table 1.** Precise tabulation of (A) The odds of developing infection by occupational categories of health care workers and (B) Association between the onset of long COVID with different risk predictors.

**Supplementary Material – Questionnaire**

Note: In cases where the question was multiple choice/dropdowns i.e. options were given, they are stated explicitly. If no options to a given question are explicitly stated, then the question was open-ended.

1. **Demographics**
2. Date of filling form:
3. Name:
4. Age:
5. Sex:

- Male
- Female
- Prefer not to say
- Other

1. Height (in cm):
2. Weight (in kg):
3. Current occupation:

- Faculty/consultant
- Resident
- Intern
- Nurse
- Helper/attendant
- Security/bouncer
- Undergraduate student
- Administrative
- Technician
- Housekeeping/class 4 workers
- Other:

1. Department:
2. Were you ever infected with SARS-CoV-2?

- Yes (confirmed by RT-PCR/ Antigen)
- No
- Yes (Symptomatic but did not confirm)

1. **Not infected**
2. Were your family/ close contacts infected:

- Yes
- No

1. **Family/Close Contacts**
2. How many members were infected:
3. Date of infection:
4. Were you vaccinated at that time:

- Yes one dose
- Yes both doses
- No

1. **Whether Vaccinated**
2. Have you now received even a single dose of vaccination:

- Yes
- No

1. **Not Vaccinated**
2. Why have you not taken the vaccine till now?:
3. **Information on Vaccination**
4. Date of first vaccine dose:
5. Date of second vaccine dose:
6. Name of vaccine:

- Covishield
- Covaxin
- Sputnik
- Other:

1. Any significant adverse effects of vaccine after five days of vaccination (thrombotic events, pulmonary embolism, deep vein thrombosis, stroke):

- Yes
- No

1. **Vaccine Induced Thrombotic Event**
2. Details of thrombotic event
3. **SARS-CoV-2 Infection**
4. Date of testing positive for SARS-COV-2:
5. Date of testing negative for SARS-COV-2:
6. Symptoms:

- Fever
- Cough
- Loss of smell
- Loss of taste
- Difficulty in breathing
- Diarrhoea
- Body ache
- Headache
- Asymptomatic
- Other:

1. Severity of fever:

- 99-100
- 100-102
- More than 102
- No fever

1. Oxygen saturation (lowest)

- More than 95
- 92-95
- Not measured
- Less than 92

1. Were you infected more than once:

- Yes
- No

1. **Subsequent SARS-CoV-2 infection**
2. Date of testing positive for SARS-COV-2:
3. Date of testing negative for SARS-COV-2:
4. Symptoms:

- Fever
- Cough
- Loss of smell
- Loss of taste
- Difficulty in breathing
- Diarrhoea
- Body ache
- Headache
- Asymptomatic
- Other:

1. Severity of fever:

- 99-100
- 100-102
- More than 102
- No fever

1. Oxygen saturation (lowest)

- More than 95
- 92-95
- Not measured
- Less than 92

1. **Hospitalization**
2. Hospitalised for COVID-19:

- Yes
- No

1. Admitted in ward or ICU:

- Ward
- ICU

1. How many days:
2. Did the patient receive: (check all that apply)

- Intubation
- Supplemental oxygen
- Remdesivir
- Tocilizumab
- Itolizumab
- Steroids
- Medicines for mucormycosis
- Fabiflu
- Oral antibiotics
- Injectable antibiotics
- Nil
- Anticoagulants
- Other

1. Long covid symptoms: (check all that apply)

- Weakness or tiredness
- Shortness of breath/ Difficulty in breathing
- Body ache/ Muscle ache
- Difficulty concentrating/confusion
- Loss of smell
- Loss of taste
- Loss of appetite
- Headache
- Trouble sleeping (frequent)
- Worry/anxiety/depression/ Low mood/not enjoying anything
- Memory loss
- Constipation
- Vertigo/dizziness/tinnitus
- Cough (persistent)
- Chest pain (pericarditis/angina)
- Palpitations
- Sore throat
- Abdominal pain
- Nausea/vomiting
- Diarrhoea
- Skin rash
- Leg pain
- Joint pain
- New onset diabetes
- New onset thyroiditis
- New onset allergies
- Hepatitis
- Dysphonia (Change in voice)
- Menstrual abnormalities
- Nil
- Other:

1. Describe in detail (number of days) in any of the above:
2. **Personal History**
3. History of smoking:

- Occasional
- Frequent
- No

1. History of tobacco chewing

- Occasional
- Frequent
- No

1. Alcohol?

- Occasional
- Frequent
- No

1. Diet

- Vegetarian
- Nonvegetarian
- Eggetarian
- Other:

1. Blood group:
2. Comorbidities: (check all that apply)

- Diabetes
- Hypertension
- Hyperthyroidism
- Hypothyroidism
- Tuberculosis
- Asthma
- COPD
- Hyperlipidaemia
- Stroke
- CVS abnormality
- Nil
